# Management Strategies of Fingertip Injuries: A Case Series

**DOI:** 10.7759/cureus.84975

**Published:** 2025-05-28

**Authors:** Sushmi Ilavarasan, Manimaran R, Koppolu Kanchana

**Affiliations:** 1 General Surgery, Sree Balaji Medical College and Hospital, Chennai, IND; 2 Plastic Surgery, Sree Balaji Medical College and Hospital, Chennai, IND

**Keywords:** fingertip injuries, functional outcomes, multidisciplinary approach, rehabilitation, surgical intervention

## Abstract

Fingertip injuries are among the most frequently encountered hand traumas in clinical settings and often result from occupational, recreational, or domestic incidents. These injuries range from simple lacerations and avulsions to more complex presentations involving severe tissue loss, exposed bone, fractures, and nail bed disruptions. Given the intricate anatomy of the fingertip, comprising soft tissue, bone, nerves, and the nail unit, these injuries can significantly compromise hand function, sensation, dexterity, and aesthetic appearance. Prompt and appropriate management is crucial to minimize complications and restore optimal hand utility. This case series documents six patients with traumatic fingertip injuries, classified according to Allen's classification system, which helps determine the extent of soft tissue and bony involvement. Surgical management strategies were tailored based on the injury type and included V-Y advancement flaps, cross-finger flaps, nail bed repair, and bone shortening with primary closure. The selection of surgical technique was guided by the goal of preserving finger length, maintaining function, and achieving satisfactory cosmetic outcomes. Postoperative evaluation focused on key outcomes such as wound healing, sensory recovery, time to return to daily activities or work, and the effectiveness of rehabilitation. Five out of six patients showed excellent recovery, resuming functional use of their hands within 3-5 weeks. One patient, however, experienced a poorer outcome due to extensive bone shortening, underscoring the limitations of certain procedures in cases of severe structural damage. This study highlights the effectiveness of a systematic, classification-based approach to fingertip injury management. It also emphasizes the importance of individualized surgical planning, early intervention, and structured rehabilitation in promoting successful outcomes.

## Introduction

Fingertip injuries refer to any trauma or damage that occurs to the end of the finger, including the skin, nail, bone, and soft tissue. These injuries are common and can range from minor cuts to more severe damage involving bone fractures or tissue loss. Fingertip injuries are among the most common hand injuries encountered in clinical practice, often resulting from occupational, recreational, or domestic accidents. These injuries can range from minor lacerations to severe tissue loss, fractures, and nail bed damage, each requiring a tailored approach to ensure optimal recovery [1]. Given the intricate anatomy of the fingertip comprising skin, soft tissue, bone, and the nail apparatus, its functional and aesthetic integrity is crucial for hand dexterity and overall hand function [2]. The complexity of fingertip injuries necessitates a systematic approach to management, which includes accurate assessment, appropriate wound care, and early intervention when necessary. While some injuries can be effectively managed conservatively with wound care and splinting, others require surgical intervention to restore structural integrity and function. Additionally, rehabilitation plays a vital role in ensuring complete recovery, preventing stiffness, and facilitating a return to daily activities. This review aims to provide a comprehensive overview of the current management strategies for fingertip injuries. By examining both conservative and surgical treatment options, rehabilitation protocols, and patient outcomes, this paper seeks to enhance clinical decision-making and improve patient care. Understanding the best practices in fingertip injury management can help healthcare professionals optimize treatment approaches, minimize complications, and improve functional recovery [3].

## Materials and methods

A series of six patients who had a history of trauma was reviewed. Different types of surgical procedures were done corresponding to the type of defect according to Allen's classification (Table 1). Functional outcome, recovery time, and rehabilitation are also discussed.

**Table 1:** Allen's classification of fingertip injuries

Type	Injury description	Treatment
Type 1	Pulp injury only without exposed bone	Full-thickness graft, V-Y flap
Type 2	Pulp and nail bed injury with exposed bone	V-Y flap, shortening and closure
Type 3	Partial loss of the distal phalanx without exposed bone	Homodigital island flap, cross-finger flap
Type 4	Involves the area proximal to the lunula and distal phalanx	Homodigital island flap, bone shortening, direct closure

This case series was conducted at Sree Balaji Medical College and Hospital in Chennai, India, and included six patients who presented with fingertip injuries due to trauma. Inclusion criteria include patients with fingertip injuries involving the distal phalanx or soft tissue of the fingertip as per Allen's classification. Both pediatric and adult patients were included. In contrast, exclusion criteria include non-traumatic fingertip conditions, such as congenital deformities or chronic dermatologic diseases. Patients with comorbidities were excluded. All patients were evaluated clinically to determine the extent and classification of their injuries, using Allen's classification system as a guide. Allen's classification was chosen over other fingertip injury classification systems since it is most widely accepted due to its simplicity and it categorizes injuries based on the level of tissue loss, which directly correlates with treatment choices and prognosis. This makes it particularly useful in clinical decision-making. The study was started on November 1, 2024, and ended on January 31, 2025. The treatment plan for each patient was based on the type and severity of the fingertip defect, with a range of surgical techniques employed, including V-Y flaps, nail bed repair, cross-finger flaps, and bone shortening with closure. The choice of procedure was tailored to restore optimal function, preserve length, and achieve satisfactory cosmetic outcomes.

One commonly used method is the V-Y advancement flap, particularly suitable for Allen's type 1 and 2 injuries involving pulp loss with or without nail bed damage. This technique involves creating a V-shaped incision in the volar pulp, which is then advanced forward to cover the defect. The incision is subsequently closed in a Y-shaped configuration, enabling tension-free closure. It is advantageous for preserving finger length and sensation, and it offers excellent aesthetic and functional results. In pediatric cases, the same principle is adapted with modifications for the smaller anatomy, ensuring optimal recovery and cosmetic outcomes. Another procedure employed is nail avulsion with figure-of-8 suturing, used in cases of nail bed injury where the nail has been partially or completely avulsed. In this technique, the avulsed nail is removed, and the nail bed is sutured using a stabilizing figure-of-8 pattern. This method ensures the nail fold and nail bed contour are preserved to support healthy regrowth of the nail plate, minimizing long-term deformities. The cross-finger flap is indicated in Allen's type 3 injuries, where there is substantial loss of pulp tissue and local flaps are insufficient for coverage. This two-stage procedure involves raising a flap from the dorsal surface of an adjacent finger and transposing it over the injured fingertip. After vascular integration, the flap pedicle is divided in a subsequent operation. Though this method immobilizes both fingers temporarily, it is highly effective in covering larger defects and restoring function. The V-Y advancement flap, while commonly used for fingertip reconstruction, has several disadvantages. It offers limited advancement, making it unsuitable for large defects or significant tissue loss. Closure under tension can compromise the blood supply, increasing the risk of flap necrosis or wound dehiscence. Additionally, the flap may result in a bulky or cosmetically unappealing fingertip, and sensory recovery may be incomplete, leading to numbness or altered sensation.

In cases where the injury involves significant damage to the nail bed, nail bed repair and suturing are performed under magnification. The nail bed is approximated with fine absorbable sutures, and either the original or an artificial nail is placed to serve as a splint. This promotes even healing of the nail bed and ensures the proper alignment of the new nail plate, reducing the risk of nail deformities. When the distal phalanx is exposed and sufficient soft tissue is not available for closure, bone shortening and primary closure are considered. This involves trimming the protruding bone to a level where soft tissues can be approximated without tension. Though this method simplifies the reconstruction, it may lead to a shortened digit and reduced functional outcome, as noted in one patient.

Each patient was followed up postoperatively for at least one month. Functional recovery, return to daily activities, and rehabilitation progress were monitored. The following test, such as the Disabilities of the Arm, Shoulder, and Hand (DASH) score, was used in the evaluation of postoperative functional recovery. Data on recovery time, postoperative complications, and final outcomes were documented and analyzed to assess the effectiveness of the chosen treatment strategies.

## Results

A total of six patients with fingertip injuries were included in this case series. All individuals had a history of trauma, with varying degrees of soft tissue and bony involvement classified according to Allen's classification. Each case was managed with a specific surgical approach tailored to the extent and nature of the injury. All patients' functional outcome was evaluated using the DASH score. The DASH score, which stands for Disabilities of the Arm, Shoulder, and Hand, is a standardized self-reported questionnaire used to evaluate the physical function and symptoms of individuals with upper limb disorders, including fingertip injuries. The statistical analysis of the DASH score is shown in Table 2.

**Table 2:** Statistical analysis of the DASH score Interpretation: Overall, the average DASH score indicates mild disability, with most patients achieving good functional recovery, except one (Case 6) with a significantly worse outcome. DASH: Disabilities of the Arm, Shoulder, and Hand

Metric	Value
Average	18.75
Minimum	0
Maximum	60
Range	0-60

Case 1

**Figure 1:**
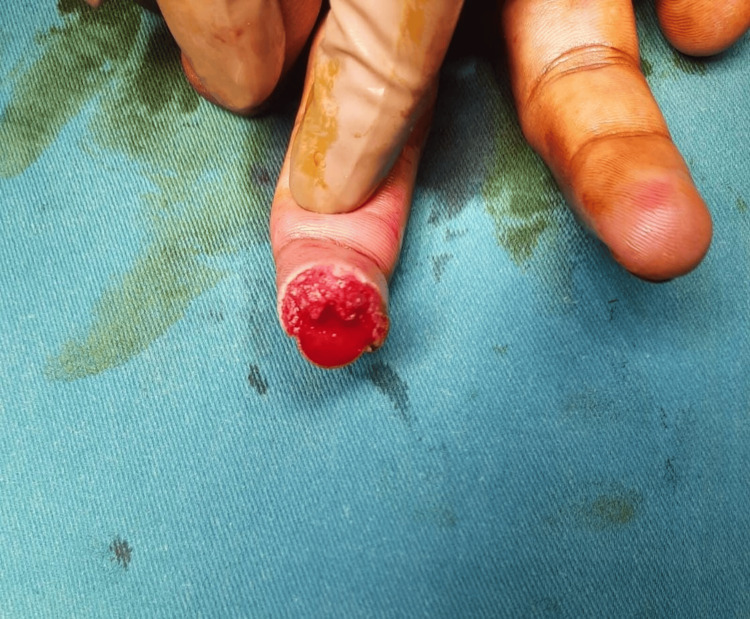
Case 1: preoperative picture showing injury over the left middle finger A 15-year-old male patient with an alleged history of trauma had an injury over the left middle finger involving a type 4 defect.

**Figure 2:**
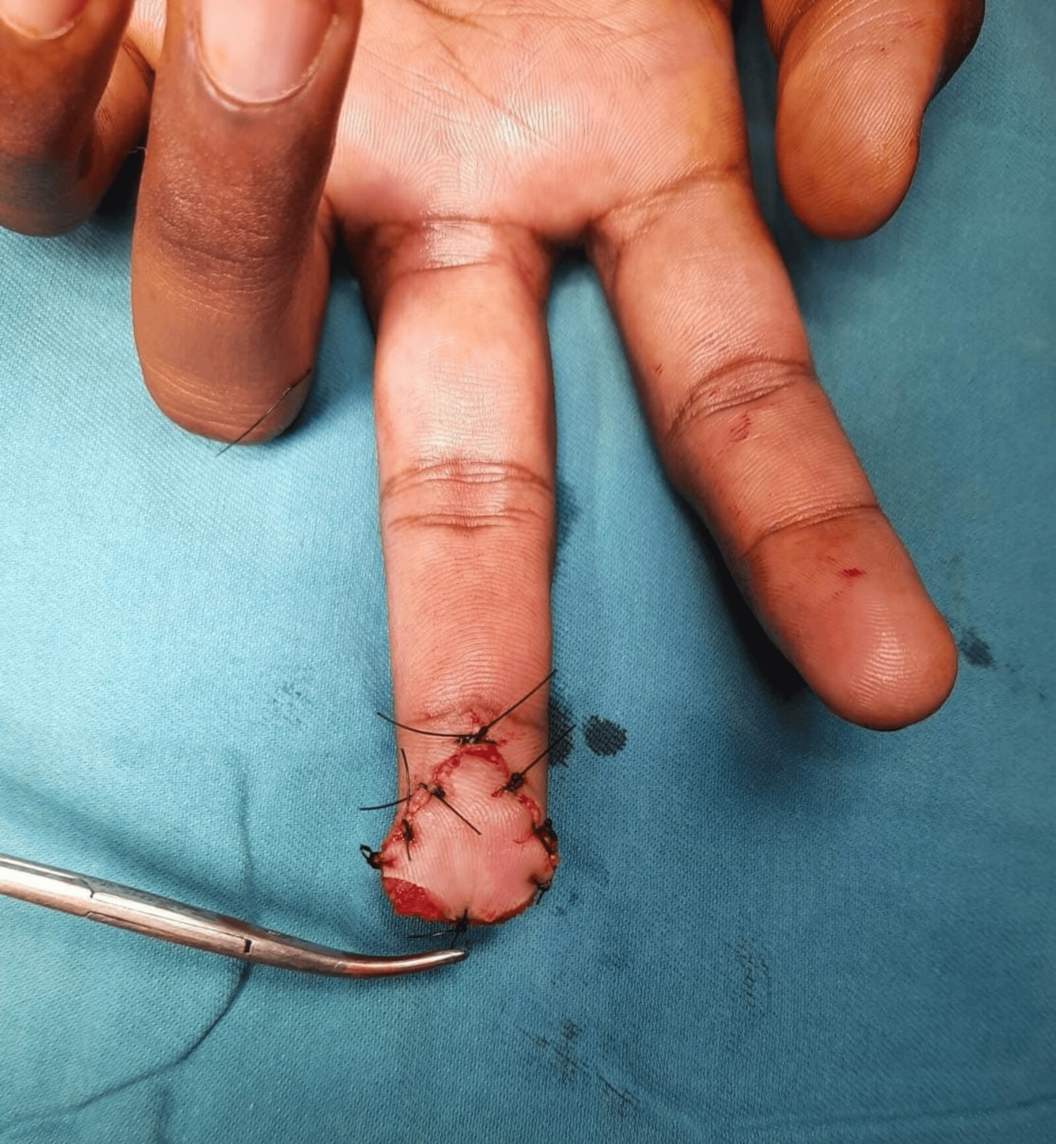
Case 1: postoperative picture showing V-Y flap The procedure done was V-Y flap. The functional outcome was good, and the average time to return to work was four weeks. Rehabilitation was done after two weeks.

The first case is a 15-year-old male patient who presented with a traumatic injury to his left middle finger, which was classified as a type 4 injury according to Allen's classification (Figure 1). This type of injury involves damage proximal to the lunula and the distal phalanx. Clinical evaluation guided the management plan, and the patient underwent a V-Y flap surgical procedure (Atasoy's flap) to repair the defect (Figure 2). Postoperative rehabilitation was initiated two weeks after surgery, focusing on restoring function and preventing stiffness. The estimated DASH score was found to be 10-15, and the functional outcome was good, with the patient able to return to his daily activities and school within four weeks. No postoperative complications were noted.

Case 2

**Figure 3:**
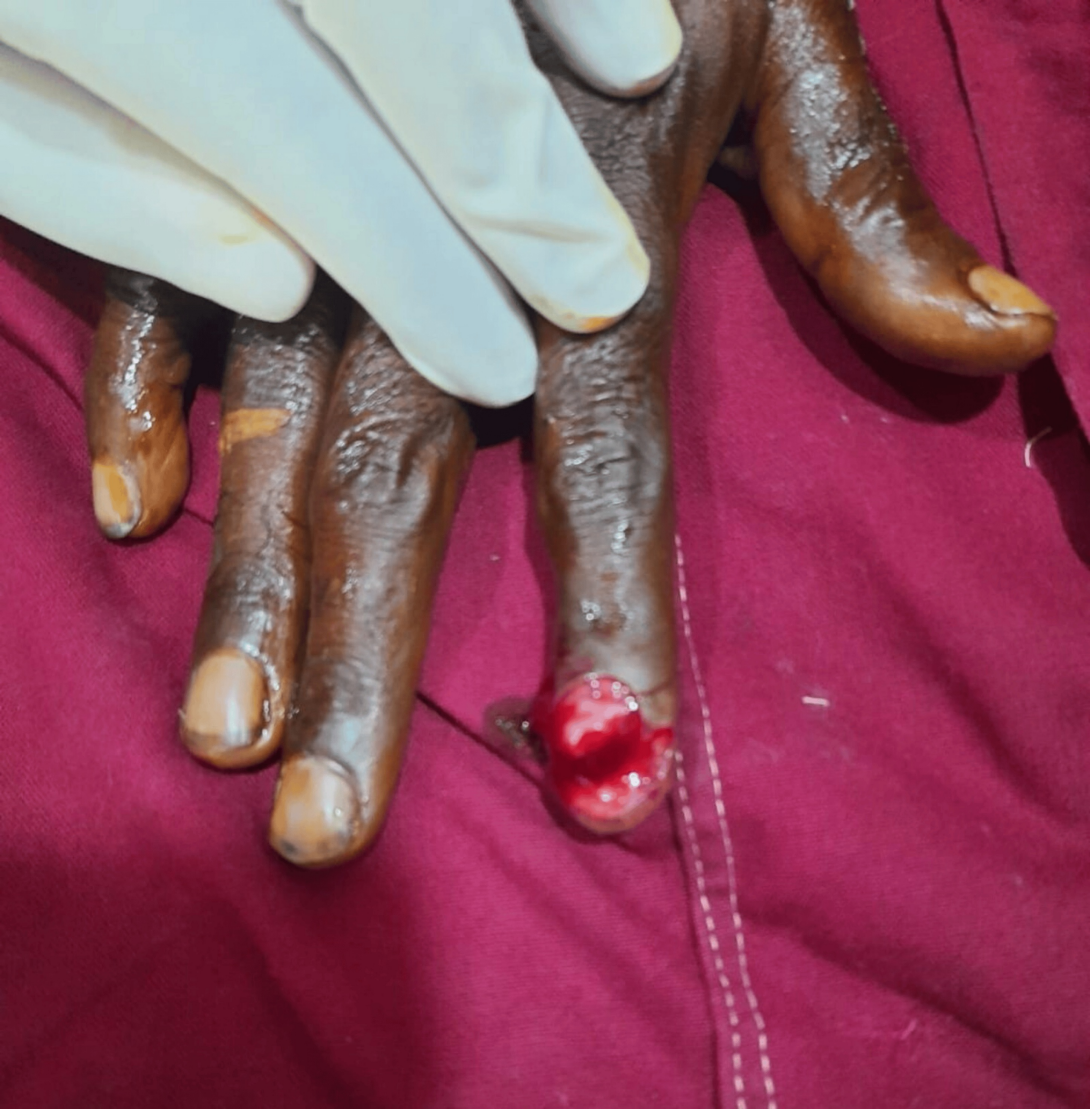
Case 2: preoperative picture showing injury over the right forefinger A 20-year-old male patient with an alleged history of trauma had an injury over the right forefinger involving a type 2 defect.

**Figure 4:**
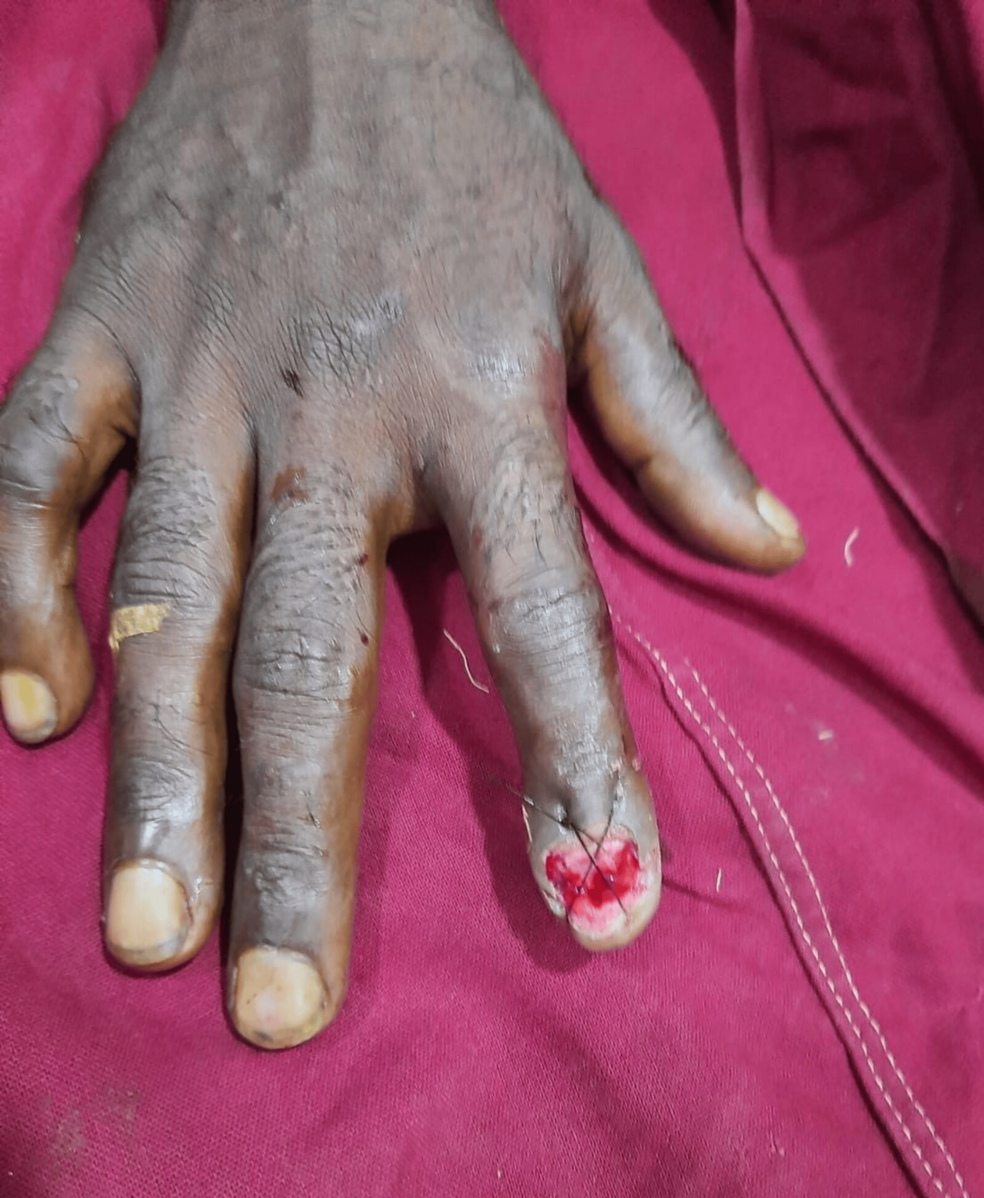
Case 2: postoperative picture showing nail avulsion with figure of 8 The procedure done was nail avulsion with figure of 8. The functional outcome was good, and the average time to return to work was four weeks. The patient was reviewed after 10 weeks with good functional outcome with minimal nail deformity.

The second case is a 20-year-old male patient who sustained trauma to the right index finger, resulting in a type 2 injury (Figure 3). This classification indicates pulp and nail bed involvement with exposed bone. The treatment approach included nail avulsion followed by figure-of-8 suturing, which is commonly used to stabilize the nail bed (Figure 4). The patient was reviewed at 10 weeks postoperatively, showing a good functional outcome with only minimal nail deformity. The estimated DASH score was found to be 10-15. He was able to return to work by week 4, and no significant complications were reported during follow-up.

Case 3

**Figure 5:**
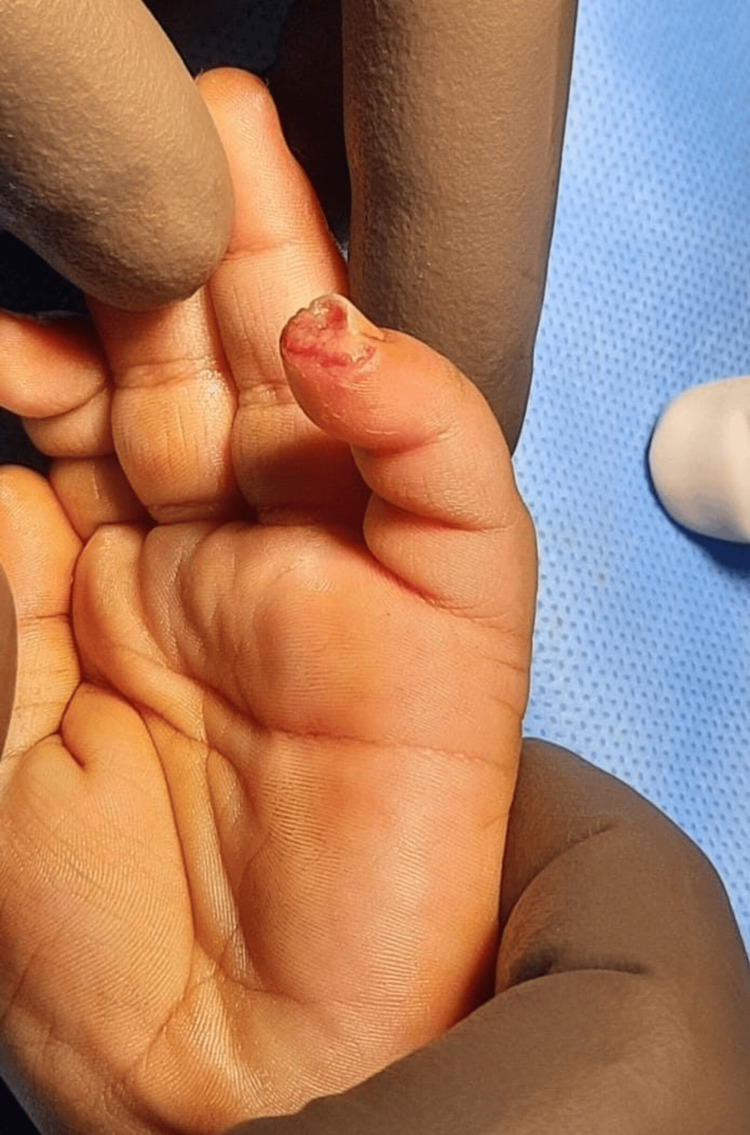
Case 3: preoperative picture showing injury over the left little finger A five-year-old boy with an alleged history of trauma had an injury over the left little finger involving a type 1 defect.

**Figure 6:**
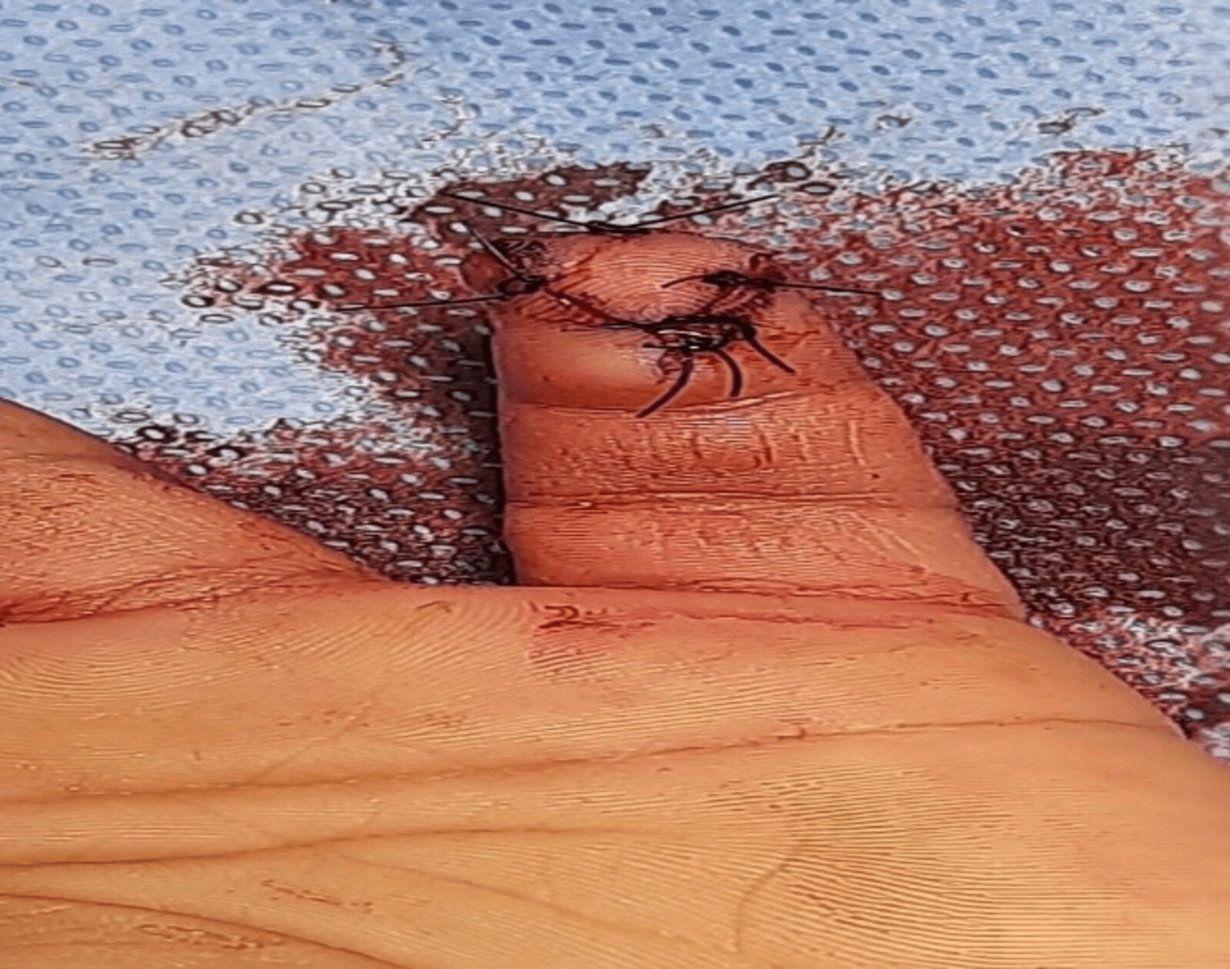
Case 3: postoperative picture showing pediatric V-Y flap The procedure done was pediatric V-Y flap. The functional outcome was good, and the average time to do daily activities was three weeks.

The third case is a five-year-old boy who suffered trauma to his left little finger, presenting with a type 1 fingertip injury, characterized by a pulp-only injury with no bone exposure (Figure 5). Due to the patient's age and the nature of the injury, a pediatric V-Y advancement flap was performed (Figure 6). Beneath the nail, the tip of the terminal phalanx was exposed on debridement; hence, a V-Y flap was performed and was not left alone to heal for secondary intention. The surgery was successful, and the child was able to resume his daily activities within three weeks. The estimated DASH score was found to be 0-5, and the functional outcome was excellent. No postoperative issues or complications were noted. Other scoring systems used in the pediatric age group are the Pediatric Outcomes Data Collection Instrument (PODCI), the Childhood Health Assessment Questionnaire (CHAQ), and the Patient-Reported Outcomes Measurement Information System (PROMIS) Pediatric Upper Extremity Scale. Rehabilitation specifics were not detailed, possibly due to the minimal nature of the injury and the rapid recovery in this pediatric patient.

Case 4

**Figure 7:**
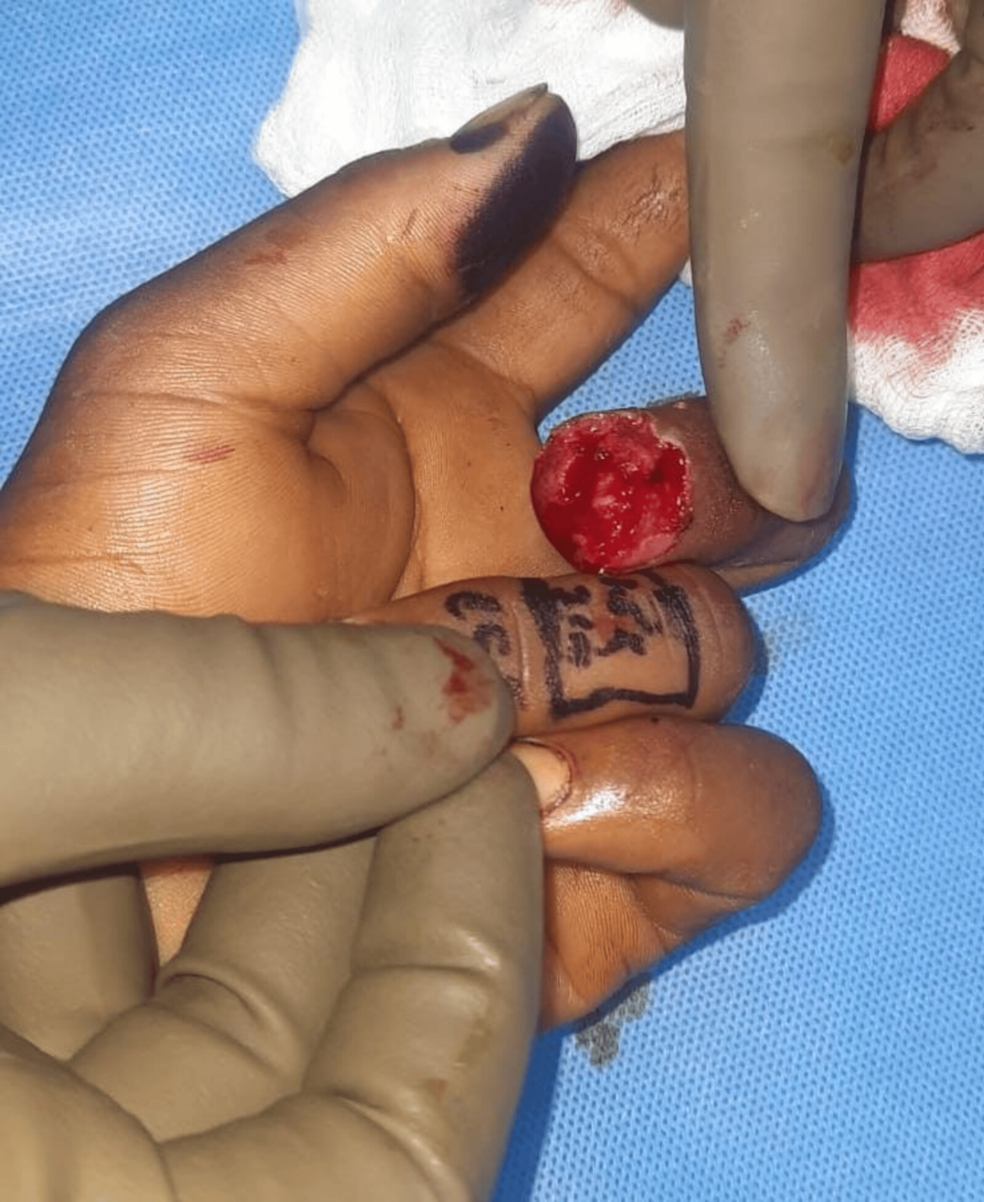
Case 4: preoperative picture showing injury over the right middle finger A 16-year-old male patient with an alleged history of trauma had an injury over the right middle finger involving a type 3 defect.

**Figure 8:**
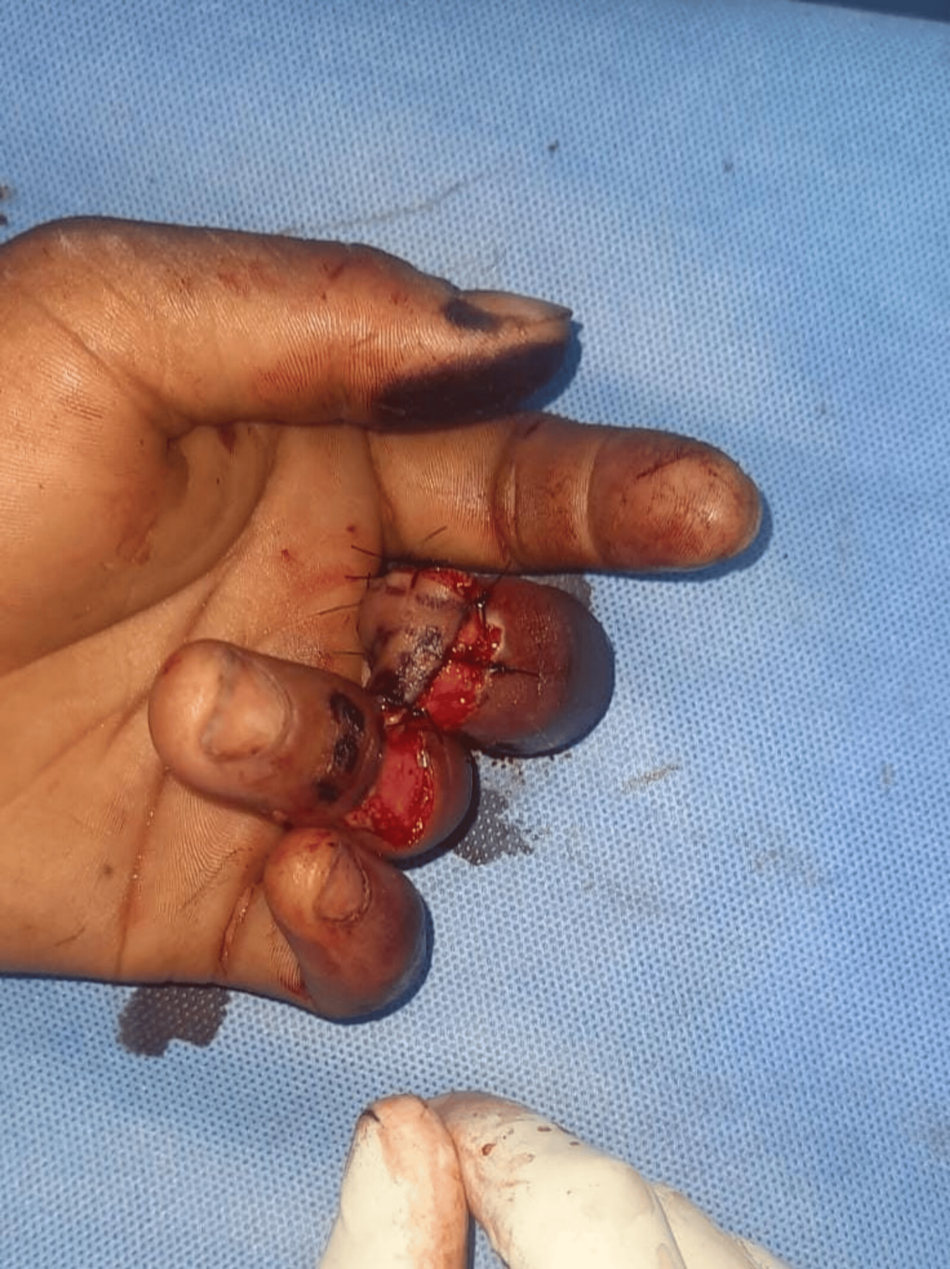
Case 4: postoperative picture showing cross-finger flap The procedure done was cross-finger flap. The functional outcome was good, and the average time to return to work was five weeks. Rehabilitation was done after two weeks.

The fourth case is a 16-year-old male patient who reported with a traumatic injury to the right middle finger, which was categorized as a type 3 injury (Figure 7). This involves partial loss of the distal phalanx without exposed bone. The surgical procedure performed was a cross-finger flap, typically used for more complex reconstructions when local tissue is insufficient (Figure 8). The duration between stage 1 and stage 2 was three weeks. Rehabilitation was started after stage 2 of the procedure. The estimated DASH score was found to be 15-20, and the functional outcome was good. The patient successfully returned to work by week 5, indicating good recovery and hand function. No complications were reported.

Case 5

**Figure 9:**
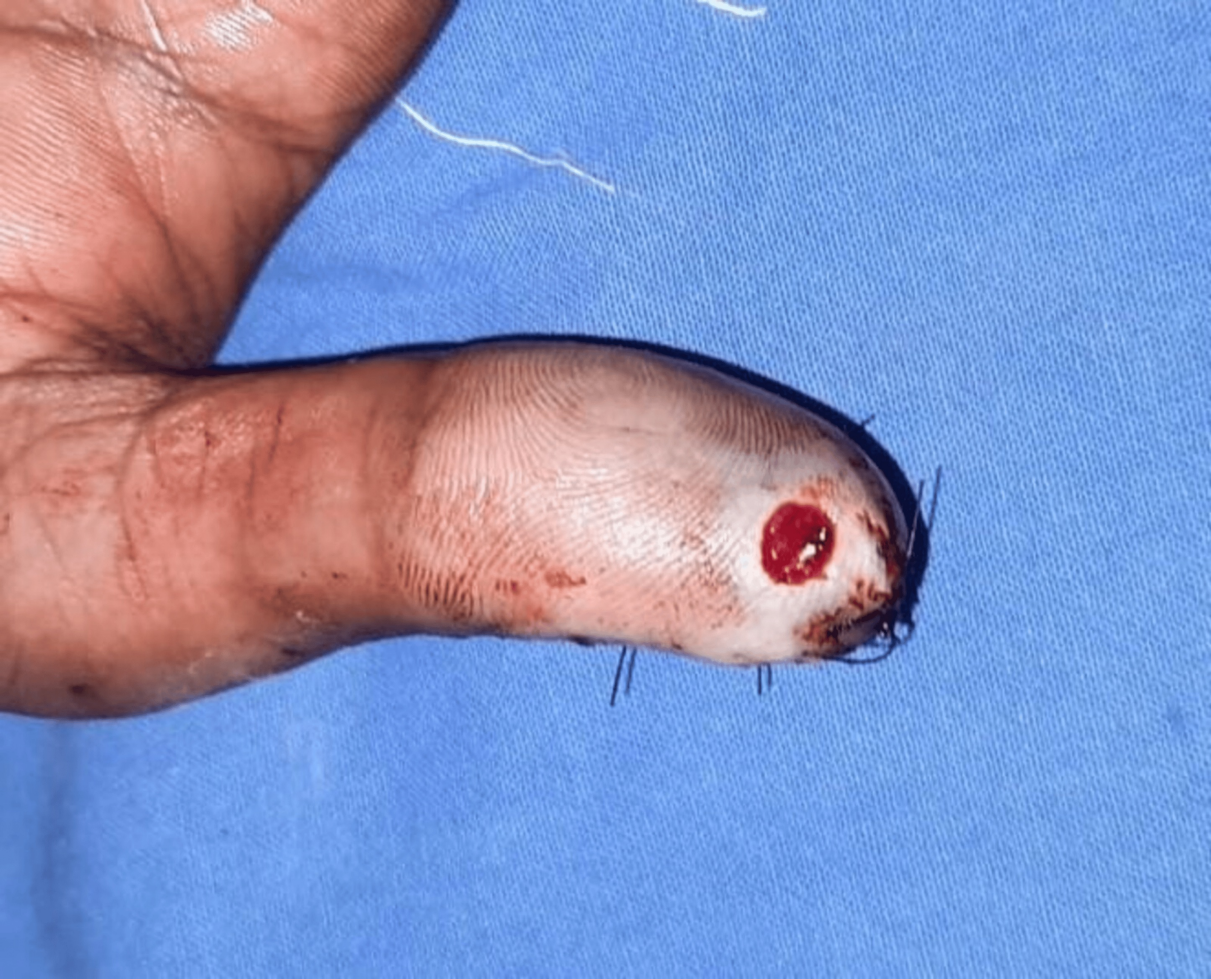
Case 5: preoperative picture showing injury over the left thumb A 23-year-old male patient with an alleged history of trauma had an injury over the left thumb involving a type 2 defect.

**Figure 10:**
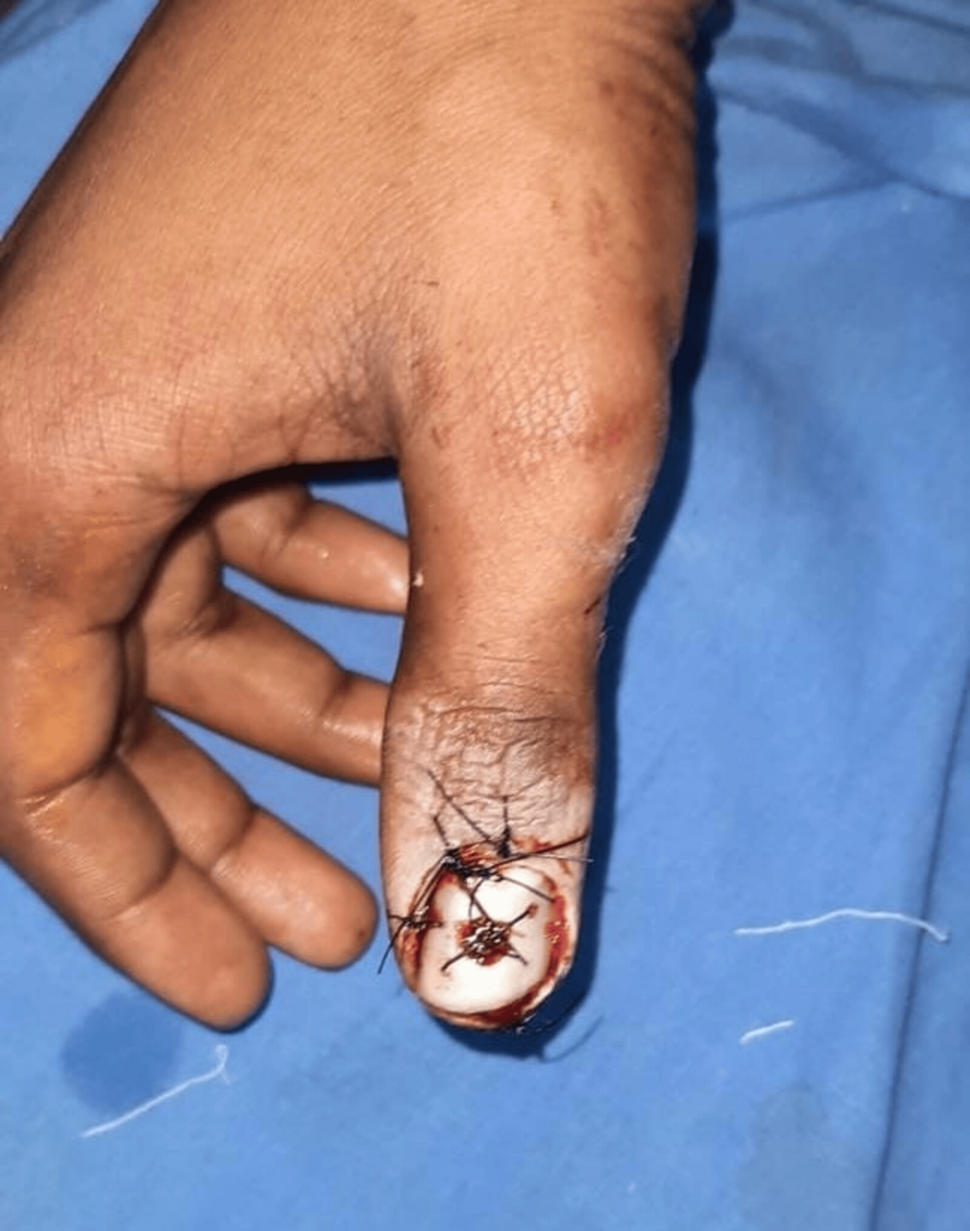
Case 5: postoperative picture showing nail fixation and suturing The procedure done was nail fixation and suturing. The functional outcome was good, and the average time to return to work was three weeks. Rehabilitation was done after two weeks.

The fifth case is a 23-year-old male patient with trauma to the left thumb, also resulting in a type 2 injury (Figure 9). Similar to Case 2, there was involvement of the pulp and nail bed with exposed bone. The surgical management included nail fixation and suturing to preserve and realign the nail bed and soft tissues (Figure 10). Rehabilitation started two weeks postoperatively. The estimated DASH score was found to be 10-15, and the functional outcome was good, resuming normal activities by week 3. No complications or deformities were noted, suggesting effective surgical and postoperative care.

Case 6

**Figure 11:**
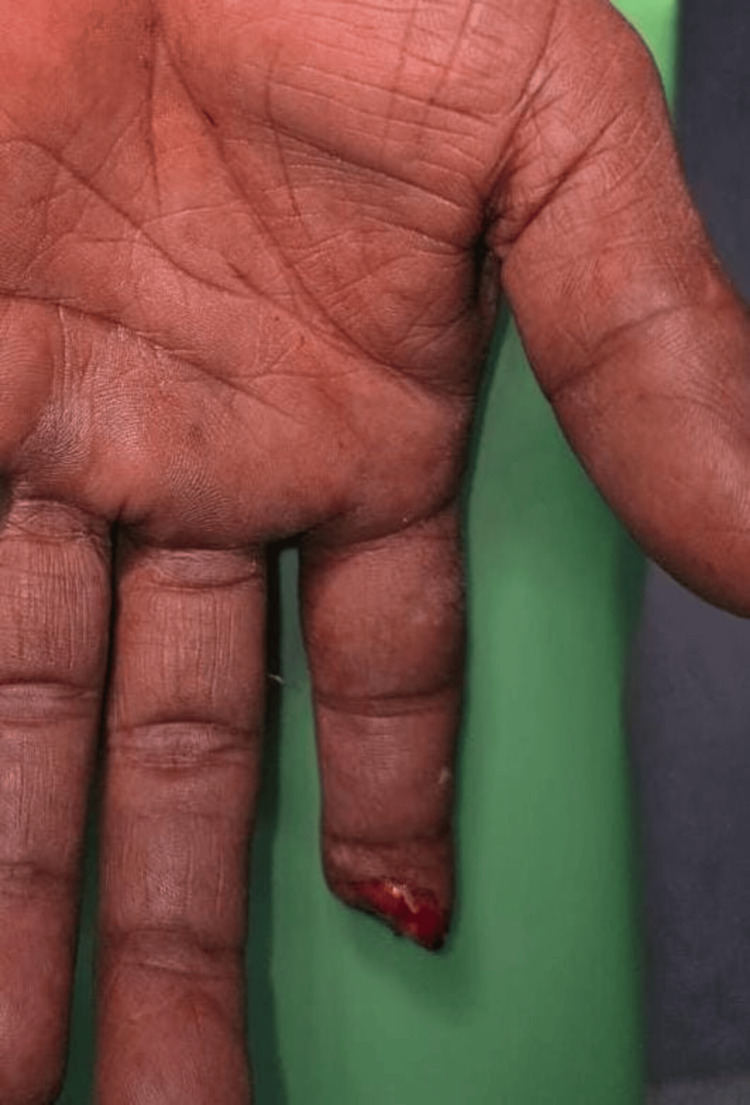
Case 6: preoperative picture showing injury over the left forefinger A 25-year-old male patient with an alleged history of trauma had an injury over the left forefinger involving a type 4 defect.

**Figure 12:**
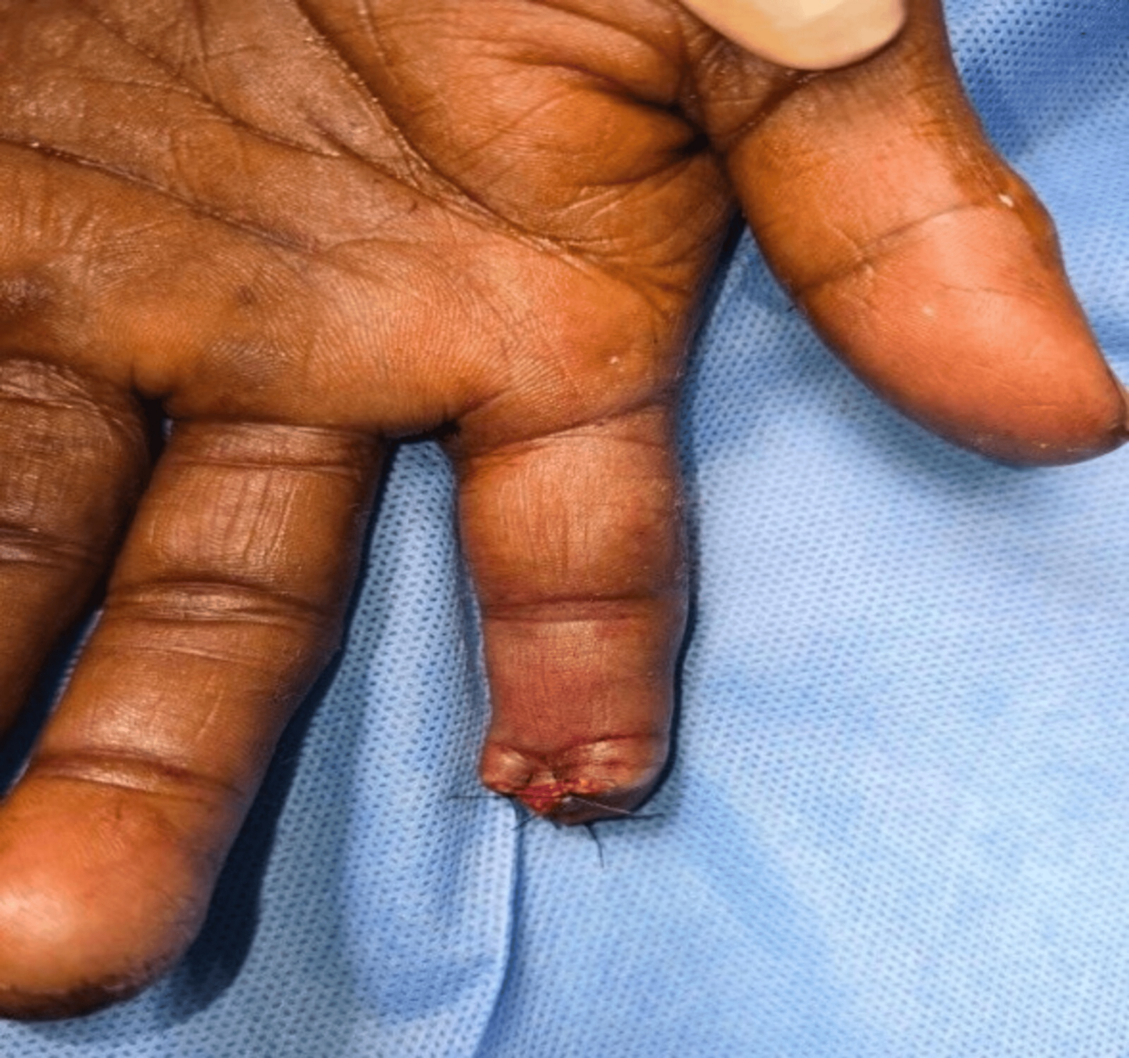
Case 6: postoperative picture showing shortening and closure The procedure done was shortening and closure. The functional outcome was poor, and the average time to return to work was four weeks.

The sixth case is a 25-year-old male patient who suffered trauma to the left index finger, classified as a type 4 injury, indicating significant tissue loss extending proximal to the lunula (Figure 11). The surgical procedure performed was bone shortening followed by primary closure, a method used when bone exposure is significant and flap reconstruction is not feasible (Figure 12). V-Y advancement flap from the radial side was not done due to extensive proximal soft tissue loss, inadequate donor tissue, and the presence of exposed bone, which precluded tension-free flap advancement. Thus, bone shortening with primary closure was chosen as a salvage option despite the risk of poor function. The estimated DASH score was found to be 50-60, and the functional outcome was poor, with notable limitations in hand function post-recovery. He returned to work after four weeks, but the irreversible nature of the injury and the extent of shortening likely contributed to the suboptimal result, making this the only case in the series with a poor prognosis.

In this case series, six male patients aged between five and 25 years presented with fingertip injuries resulting from trauma, classified using Allen's system, ranging from type 1 to type 4. Each case was managed surgically with procedures tailored to the severity and type of injury. Two patients with type 4 injuries (Cases 1 and 6) underwent V-Y flap and bone shortening with closure; while Case 1 had a good outcome, Case 6 showed poor functional recovery due to irreversible tissue loss. Two patients with type 2 injuries (Cases 2 and 5) were treated with nail avulsion with figure-of-8 suturing and nail fixation with suturing, both resulting in good outcomes with minimal complications. A five-year-old child with a type 1 injury (Case 3) recovered well after a pediatric V-Y flap, returning to normal activities in three weeks. Case 4, with a type 3 injury, was managed with a cross-finger flap and also had a positive recovery. In five out of six cases, patients returned to daily activities or work within 3-5 weeks, supported by rehabilitation initiated around two weeks post-surgery. Only one patient experienced a poor outcome, highlighting the importance of injury severity and surgical choice in determining recovery success (Table 3).

**Table 3:** Comparative analysis of demographic profiles and clinical assessments of all six patients

Case	Age/sex	Socioeconomic background	Cause of injury	Type of injury (Allen's classification)	Diagnostic tests	Surgical procedure	Complications	Functional outcome	Recovery time
1	15/male	Lower class	Trauma	Type 4: proximal to the lunula	Clinical examination and X-ray of the hand	V-Y flap	None reported	Good	Return to work in 4 weeks
2	20/male	Lower class	Trauma	Type 2: pulp and nail bed with exposed bone	Clinical examination and X-ray of the hand	Nail avulsion with figure-of-8 suture	Minimal nail deformity	Good	Return to work in 4 weeks
3	5/male	Lower class	Trauma	Type 1: pulp injury only	Clinical examination and X-ray of the hand	Pediatric V-Y flap	None reported	Good	Return to daily activities in 3 weeks
4	16/male	Lower class	Trauma	Type 3: partial distal phalanx loss	Clinical examination and X-ray of the hand	Cross-finger flap	None reported	Good	Return to work in 5 weeks
5	23/male	Lower class	Trauma	Type 2: pulp and nail bed with exposed bone	Clinical examination and X-ray of the hand	Nail fixation and suturing	None reported	Good	Return to work in 3 weeks
6	25/male	Lower class	Trauma	Type 4: proximal to the lunula	Clinical examination and X-ray of the hand	Bone shortening and closure	None reported	Poor	Return to work in 4 weeks

Overall, this case series highlighted that surgical intervention combined with a structural rehabilitation plan contributed to positive functional outcomes in most patients.

## Discussion

Limitations

Small Sample Size

The study is based on a case series of only six patients, which limits the generalizability of the findings. A larger sample would provide more robust and statistically significant insights into treatment efficacy and outcomes.

Lack of Control Group

The study does not include a comparison or control group. Without a standardized baseline or comparison to other treatment modalities, it is difficult to objectively assess the relative effectiveness of each management strategy.

Heterogeneity of Treatment Modalities

Different surgical techniques were used for different types of injuries based on Allen's classification. This variability in procedures makes it challenging to draw uniform conclusions about the success of any one method.

Short-Term Follow-Up

The study does not detail long-term follow-up outcomes, particularly regarding nail growth, sensory recovery, chronic pain, or functional restoration. These are critical for evaluating the true success of fingertip injury management.

Single-Center Experience

The study reflects the experience of a single institution or surgical team, which may limit external validity and applicability across different clinical settings.

Potential Bias

Being a retrospective case series, the study is inherently prone to selection and observational bias, especially as it lacks blinding or randomization.

Management strategies

Conservative management of fingertip injuries focuses on wound care, pain relief, infection prevention, and functional recovery. The wound should be thoroughly cleaned with saline to remove debris and reduce the risk of infection. A non-adherent dressing, such as paraffin gauze or hydrogel, should be applied to keep the wound moist, followed by a sterile compressive bandage. Dressings should be changed regularly to maintain cleanliness and promote healing [4]. Pain can be managed with acetaminophen or nonsteroidal anti-inflammatory drugs (NSAIDs), while severe pain may require short-term opioid use [5]. Infection control is essential, with tetanus prophylaxis administered if indicated. Prophylactic antibiotics may be considered for contaminated, bite, or crush injuries. Hemostasis can usually be achieved with direct pressure, though hemostatic agents may be used if necessary [6]. For nail bed injuries, a subungual hematoma larger than 50% of the nail should be drained via trephination, while a partially avulsed but viable nail should be preserved as a splint. If joint involvement is suspected, a splint may be applied to immobilize the finger to prevent further damage and aid in healing. Activity modification is advised, and a follow-up should be scheduled within 7-10 days. Referral is necessary for exposed bone, severe nail bed injuries, vascular compromise, or suspected fractures [7].

Fingertip injuries are common and require careful surgical management to restore function, sensation, and aesthetics. Injuries with a fractured distal phalanx with no tissue loss can be managed conservatively; splinting or suturing can be done. The approach depends on the extent of soft tissue loss, bone exposure, and vascular compromise. Simple lacerations or partial-thickness injuries may be managed with primary closure or local wound care. When significant tissue loss occurs, reconstructive options include skin grafts, local flaps, or composite tissue transfer. V-Y advancement flaps or Kutler flaps are effective for volar defects, while cross-finger flaps are useful for larger defects [8,9]. The disadvantages of V-Y advancement flaps are as follows: it offers limited advancement, making it unsuitable for large injuries or those extending proximal to the lunula. In cases with significant bone exposure, the flap may not provide adequate coverage or volume. High-tension closures can lead to flap edge necrosis or dehiscence, and in crush injuries, compromised local vascularity increases the risk of flap failure. Additionally, some patients may experience sensory disturbances due to nerve stretch, and the resulting Y-shaped scar may pose cosmetic concerns. These drawbacks make the V-Y flap less ideal for extensive, proximal, or poorly vascularized fingertip injuries. The second-layer palmar graft is a surgical procedure done for reconstructing fingertip defects, providing an excellent aesthetic appearance in terms of color match and optimal function. Reverse homodigital island flaps are increasingly used to reconstruct traumatic fingertip injuries [10]. The advantages of the reverse homodigital island flap include its single-stage implementation (as opposed to multistage regional flaps), resulting in shorter hospital stays, an earlier return to work, and diminished associated costs. For injuries with exposed bone, shortening and primary closure may be considered if functional impairment is minimal. Replantation is an option in select cases, particularly in children or when the amputated segment is viable [11]. The choice of surgical technique depends on injury severity, patient factors, and the goal of optimizing hand function. Postoperative care, including immobilization, wound care, and rehabilitation, is crucial to prevent stiffness and improve outcomes. Early intervention with an appropriate surgical approach can significantly enhance recovery, reduce complications, and restore the patient's ability to perform daily activities effectively [12]. With the advancement of microsurgical techniques, free tissue transfer using the toe pulp, particularly via mini hallux toenail flaps pedicled with the hallux transverse artery and toe pulp vein, has emerged as an effective and aesthetic option for fingertip reconstruction [13].

Rehabilitation plays a crucial role in the management of fingertip injuries, ensuring optimal functional recovery, minimizing complications, and restoring hand dexterity. The rehabilitation process begins immediately after surgical or conservative treatment to prevent stiffness, hypersensitivity, and contractures. Rehabilitation protocol involves early initiation of rehabilitation, gradual mobilization, desensitization techniques, sensory re-education, strengthening exercises, scar and wound management, occupational therapy, psychological and social support, follow-up, and monitoring. Immobilization is initially required for wound healing and protection, typically using splints or dressings [14]. However, prolonged immobilization can lead to joint stiffness, so early mobilization is encouraged as soon as healing permits. Range of motion exercises, including gentle active and passive movements, help maintain flexibility and prevent tendon adhesions [15]. Desensitization techniques, such as graded exposure to different textures and temperatures, are essential in managing post-injury hypersensitivity, which is common in fingertip injuries. Sensory re-education is also beneficial, especially in cases where nerve damage has occurred, helping patients regain proper tactile perception [16]. Strengthening exercises, including grip and pinch training, improve hand function and prevent muscle atrophy. Occupational therapy may be required for individuals with significant functional limitations, focusing on task-specific training to facilitate daily activities [17]. Proper wound care, scar management with massage and silicone therapy, and pain management strategies further support recovery [18]. Psychological support may also be needed, as fingertip injuries can impact a patient's ability to perform routine tasks, leading to frustration or anxiety. Regular follow-ups with a hand therapist or specialist ensure progress is monitored and complications are addressed early [19]. A structured and individualized rehabilitation program enhances recovery, helping patients regain optimal hand function and return to work or daily activities with minimal impairment [20].

## Conclusions

Regardless of the treatment approach, rehabilitation through early mobilization, splinting, and occupational therapy plays a crucial role in restoring hand function and preventing long-term stiffness. Patient outcomes depend on the timeliness and appropriateness of treatment, with evidence suggesting that a multidisciplinary approach involving surgeons, therapists, and primary care providers yields the best results. The psychosocial impact of fingertip injuries, including concerns about aesthetics and functionality, is also essential for comprehensive patient care. By integrating evidence-based practices and a patient-centered approach, healthcare professionals can improve clinical decision-making and enhance the overall recovery process. Comprehensive care that considers both physical and psychosocial aspects is essential to improve the quality of life for patients recovering from fingertip trauma. Continued research and advancements in treatment modalities will further refine management strategies, ultimately leading to better functional and psychological outcomes for patients with fingertip injuries.

## References

[1] Pencle F, Doehrmann R, Waseem M (2025). Fingertip injuries. StatPearls [Internet].

[2] Satku M, Puhaindran ME, Chong AK (2015). Characteristics of fingertip injuries in children in Singapore. Hand Surg.

[3] Evans DM, Bernardis C (2000). A new classification for fingertip injuries. J Hand Surg Br.

[4] Fassler PR (1996). Fingertip injuries: evaluation and treatment. J Am Acad Orthop Surg.

[5] Peterson SL, Peterson EL, Wheatley MJ (2014). Management of fingertip amputations. J Hand Surg Am.

[6] Schaefer E, Lawson J, Ibrahim T, Yohe G, Zhang G, Giladi AM (2023). Antibiotic prophylaxis in the management of distal fingertip amputation and crush injury. J Hand Surg Glob Online.

[7] George A, Alexander R, Manju C (2017). Management of nail bed injuries associated with fingertip injuries. Indian J Orthop.

[8] Kawaiah A, Thakur M, Garg S, Kawasmi SH, Hassan A (2020). Fingertip injuries and amputations: a review of the literature. Cureus.

[9] Rubin G, Orbach H, Rinott M, Wolovelsky A, Rozen N (2015). The use of prophylactic antibiotics in treatment of fingertip amputation: a randomized prospective trial. Am J Emerg Med.

[10] Bindra RR (1996). Management of nail-bed fracture-lacerations using a tension-band suture. J Hand Surg Am.

[11] de Putter CE, Selles RW, Polinder S, Panneman MJ, Hovius SE, van Beeck EF (2012). Economic impact of hand and wrist injuries: health-care costs and productivity costs in a population-based study. J Bone Joint Surg Am.

[12] Kim KS, Kim ES, Hwang JH, Lee SY (2013). Fingertip reconstruction using the hypothenar perforator free flap. J Plast Reconstr Aesthet Surg.

[13] Fan X, Zhou Y, Zhou J, Dai S, Liu J, Lao K (2023). The reconstruction of fingertip injury by mini hallux toenail flap pedicled with the hallux transverse artery and toe pulp vein transplantation technique based on the equivalent design theory. BMC Surg.

[14] Altergott C, Garcia FJ, Nager AL (2008). Pediatric fingertip injuries: do prophylactic antibiotics alter infection rates?. Pediatr Emerg Care.

[15] Patil RK, Koul AR (2012). Early active mobilisation versus immobilisation after extrinsic extensor tendon repair: a prospective randomised trial. Indian J Plast Surg.

[16] Xia W, Bai Z, Dai R, Zhang J, Lu J, Niu W (2021). The effects of sensory re-education on hand function recovery after peripheral nerve repair: a systematic review. NeuroRehabilitation.

[17] Hubbard IJ, Parsons MW, Neilson C, Carey LM (2009). Task-specific training: evidence for and translation to clinical practice. Occup Ther Int.

[18] Khansa I, Harrison B, Janis JE (2016). Evidence-based scar management: how to improve results with technique and technology. Plast Reconstr Surg.

[19] Meyer TM (2003). Psychological aspects of mutilating hand injuries. Hand Clin.

[20] Loeffler BJ, Lewis DR (2016). Restoration of elbow flexion. Hand Clin.

